# The Great Mimicker in Bone: A Rare Case of Melioidosis With Multifocal Skeletal Manifestations and Diagnostic Challenges

**DOI:** 10.7759/cureus.108304

**Published:** 2026-05-05

**Authors:** Madhumita Sugumaran, Minmini Selvam, Rajoo Ramachandran, Prerna Agarwal, Venkata Sai, Prithvi Sudhakar, Niranjana Sugumaran

**Affiliations:** 1 Department of Radiology, Sri Ramachandra Institute of Higher Education and Research, Chennai, IND

**Keywords:** burkholderia pseudomallei, diabetes mellitus, great mimicker, melioidosis, multifocal skeletal disease, osteomyelitis, septic arthritis

## Abstract

Melioidosis, caused by the Gram-negative bacillus *Burkholderia pseudomallei *(*B. pseudomallei*), is an emerging infectious disease endemic to Southeast Asia and northern Australia, well known for its protean manifestations and aptly termed “the great mimicker.” Skeletal involvement, although an established entity, remains underreported, particularly when affecting multiple bones simultaneously in the setting of newly diagnosed diabetes mellitus. We report a 35-year-old male patient with newly diagnosed type II diabetes mellitus who presented with fever and right knee pain. Surgical debridement of a right distal thigh abscess with bone biopsy yielded *B. pseudomallei* on culture. Subsequent imaging revealed multifocal skeletal disease involving the right distal femur, bilateral knees, left heel (calcaneum, talus, distal tibia), and cervical vertebrae (C2-C6), accompanied by pulmonary septic emboli. The patient required two surgical interventions - right thigh debridement and left knee arthrotomy - and was managed with intravenous meropenem during the intensive phase, with intravenous co-trimoxazole added as an adjunct, resulting in gradual clinical improvement. This case highlights the diagnostic complexity of skeletal melioidosis in a diabetic patient presenting simultaneously with multifocal osteomyelitis, septic arthritis, pulmonary involvement, and possible spinal disease. A high index of clinical suspicion, early microbiological culture, and prompt targeted antibiotic therapy are essential to prevent mortality and morbidity in such cases.

## Introduction

Melioidosis is an infectious disease caused by *Burkholderia pseudomallei *(*B. pseudomallei*), a Gram-negative, aerobic saprophytic bacillus found in soil and surface water in tropical and subtropical regions. The disease is endemic to Southeast Asia - particularly Thailand - and northern Australia, but emerging reports from India, Africa, and South America signal a broadening geographic distribution [1,2]. Infection typically occurs through percutaneous inoculation, inhalation, or ingestion of contaminated material, with diabetes mellitus being the most important predisposing risk factor, accounting for up to 60% of cases in endemic regions [3].

The clinical spectrum of melioidosis is remarkably wide, ranging from subclinical infection to fulminant bacteremic sepsis with multi-organ failure. Pulmonary involvement is the most common presentation, but the disease can affect virtually any organ system, earning it the apt moniker “the great mimicker” [4]. Musculoskeletal manifestations, including osteomyelitis and septic arthritis, are a recognized but infrequent presentation, reported in approximately 4-14% of cases in large series [5,6]. The knee is the most commonly involved joint, followed by the ankle, hip, and shoulder [6]. Multifocal skeletal involvement with simultaneous pulmonary and possible spinal disease in a newly diagnosed diabetic represents an exceptionally challenging diagnostic and therapeutic scenario.

We present a case of a 35-year-old male patient with newly diagnosed type II diabetes mellitus who was ultimately diagnosed with multifocal skeletal melioidosis - involving the right distal femur, bilateral knees, left ankle complex (distal tibia, calcaneum, and talus), and cervical spine, along with pulmonary septic emboli - underscoring the importance of maintaining a high index of suspicion for this disease in appropriate clinical and geographic contexts.

## Case presentation

Clinical history and initial presentation

A 35-year-old male patient presented to the orthopedics department with a two-week history of right knee pain. He reported two episodes of high-grade fever treated at an outside facility prior to referral. On October 18, 2025, he developed a relapse of fever with right knee pain and was admitted to an outside hospital, where he received intravenous ceftriaxone and meropenem. Magnetic resonance imaging (MRI) of the right knee and thigh performed elsewhere demonstrated osteomyelitis of the right distal femur with an abscess in the right distal thigh.

His past medical history was significant for newly diagnosed type II diabetes mellitus. There was no history of trauma, weight loss, or anorexia. Family history was non-contributory.

Physical examination 

On admission, the patient was febrile (temperature 103°F), with pulse rate 98/min, respiratory rate 20/min, and blood pressure 130/70 mmHg. Weight was 75 kg. There was no pallor, icterus, cyanosis, clubbing, lymphadenopathy, or thyromegaly. Cardiovascular and abdominal examinations were unremarkable. Respiratory examination revealed reduced air entry in bilateral infraaxillary regions and oxygen saturation of 85% on room air, for which supplemental oxygen was immediately initiated with subsequent improvement in saturation.

Local examination of the right lower limb revealed diffuse swelling, local warmth, and tenderness over the distal one-third of the thigh and knee. Range of motion at the right knee was restricted from five to 45 degrees with pain. Distal pulses and sensation were intact. The left lower limb was initially unremarkable, with a full painless range of motion at the hip, knee, and ankle. Bilateral upper limbs and spine were clinically normal.

Investigations

Laboratory investigations revealed a total leucocyte count of 15,000 cells/µL (reference range: 4,000-11,000 cells/µL), consistent with an infective process. Blood sugar was elevated, confirming newly diagnosed diabetes mellitus. Other baseline investigations are summarized in the clinical record.

**Figure 1:**
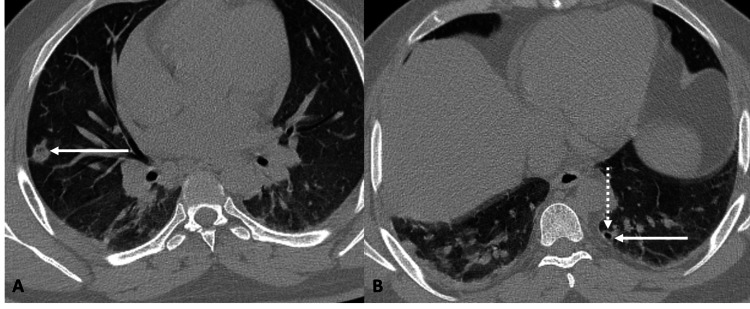
High-resolution computed tomography (HRCT) of the thorax A & B: Axial sections of the thorax in the lung window. Two nodules (solid white arrows), with one of them showing cavitation (dotted white arrow), are predominantly in the peripheral and subpleural region of bilateral lung fields.

High-resolution computed tomography (HRCT) of the thorax demonstrated multiple nodules, some with cavitation, in the anterior segment of the right upper lobe, lateral segment of the right middle lobe, anterior segment of the left upper lobe, and posterior basal segment of the right lower lobe. No pleural or pericardial effusion was identified. These findings were consistent with septic emboli or an infective etiology (Figure 1).

**Figure 2:**
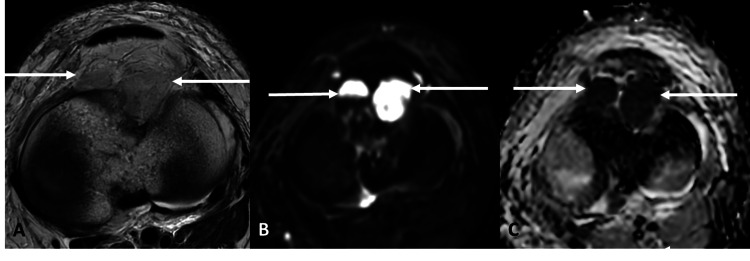
MRI of the left knee joint A, B, & C: Axial sections of the knee joint in T2 (A), DWI (B), and ADC (C) sequences. Two relatively well-defined fluid collections (solid white arrows) show restricted diffusion with corresponding ADC drop noted in the anterior aspect of the knee joint. DWI: diffusion-weighted imaging; ADC: apparent diffusion coefficient

**Figure 3:**
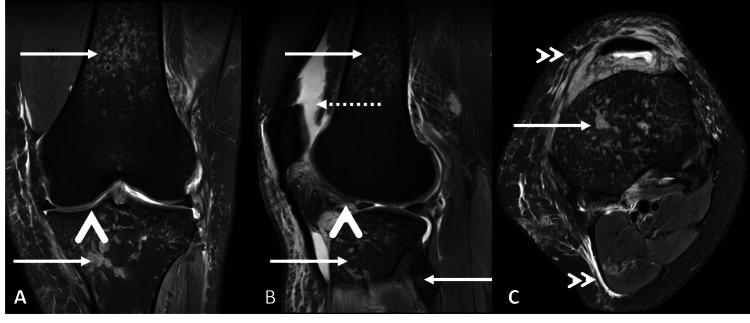
MRI of the left knee joint A, B, & C: PDFS sequence in coronal (A), sagittal (B), and axial (C) planes of the knee joint and distal thigh. Geographical areas of altered marrow signal intensities are noted in the distal shaft of the femur, proximal tibia, and fibula (solid white arrow). Moderate knee joint effusion noted extending into the suprapatellar recess (dotted white arrow). Severe reduction in the joint space with mild synovial thickening is noted (arrowhead). Extensive soft tissue edema surrounding the knee joint and distal thigh (double arrowhead). PDFS: proton density fat-saturated

MRI of the left knee, performed four days after the HRCT thorax, demonstrated two well-defined fluid collections with restricted diffusion in the anterior aspect of the knee joint (the largest measuring 2.0 × 1.8 × 1.8 cm), surrounding fat stranding, and edema in the prefemoral and Hoffa’s fat pads. Geographic areas of altered marrow signal were noted in the distal femoral shaft, proximal tibia, and neck of fibula. Moderate knee joint effusion with mild synovial thickening was identified. The anterior cruciate ligament showed proton density fat-saturated (PDFS) hyperintensity in its mid-substance. Imaging features were consistent with septic arthritis of the left knee with osteomyelitis of the distal femur, proximal tibia, and fibula (Figures 2, 3).

**Figure 4:**
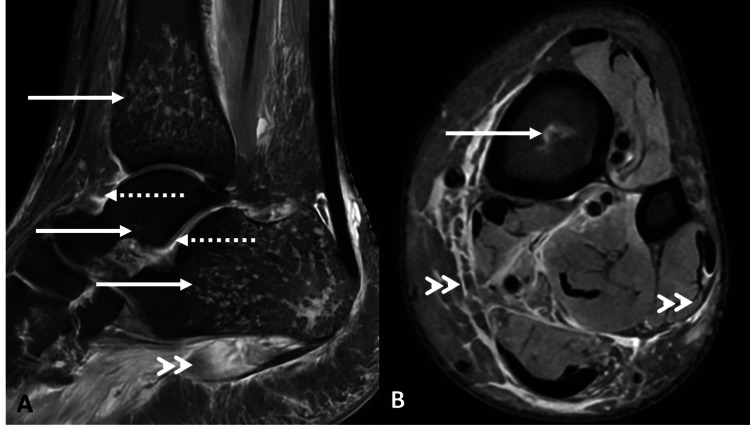
MRI of the left ankle joint A & B: PDFS sequence in sagittal (A) and axial (B) planes of the ankle joint and distal leg. Geographical areas of altered marrow signal intensities noted involving the distal aspect of tibia, calcaneum, and talus (solid white arrow). Minimal ankle joint and subtalar joint effusion (dotted white arrow). Extensive soft tissue edema surrounding the ankle joint and distal leg (double arrowhead). PDFS: proton density fat-saturated

MRI of the left ankle joint, performed one week after the HRCT thorax, demonstrated geographic areas of altered marrow signal intensity involving the distal tibia, calcaneum, and talus (medial aspect of neck and body). Diffuse subcutaneous and intramuscular edema was noted predominantly in the medial aspect of the leg and plantar aspect of the foot. Minimal ankle and subtalar joint effusion was identified. Findings were consistent with acute osteomyelitis involving the left distal tibia, calcaneum, and talus (Figure 4).

**Figure 5:**
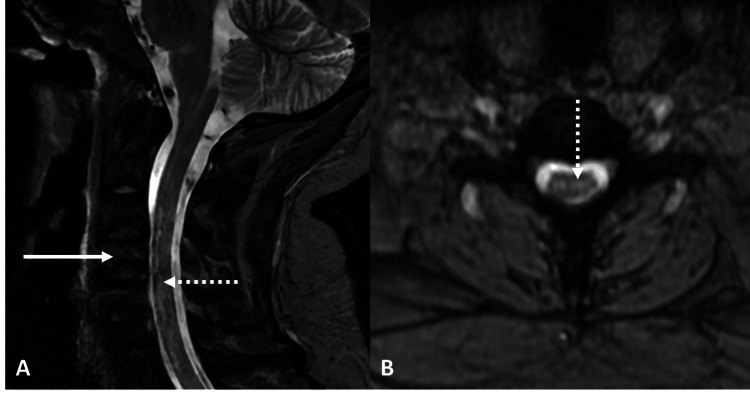
MRI of the cervical spine A & B: Geographical areas of T1 hypo and T2-weighted short tau inversion recovery (T2/STIR) hyperintensity (solid white arrow) noted involving the body of C2-C6 vertebrae. No evidence of involvement of posterior elements. Patchy areas of T2/ STIR hyperintensity (dotted white arrow) are noted in the cervical spinal cord adjacent to it.

MRI of the cervical spine, performed one week after the HRCT thorax, was obtained in view of evolving multifocal skeletal disease. This study was suboptimal due to motion artifacts but demonstrated geographic areas of T1 hypointensity and T2-weighted short tau inversion recovery (T2/STIR) hyperintensity involving the vertebral bodies of C2 through C6, with patchy T2/STIR hyperintensity in the adjacent cervical spinal cord. No posterior element involvement or epidural collection was identified (Figure 5). These findings were noted to possibly represent motion or CSF pulsation artifacts; however, given the known multifocal skeletal disease, contrast-enhanced MRI of the spine was recommended for further characterization. Plain MRI of the brain revealed no significant intracranial abnormality.

Microbiological diagnosis

Pus obtained from the right distal thigh abscess during surgical drainage yielded *B. pseudomallei *on culture, confirming the diagnosis of melioidosis. Bone marrow culture from the right distal femur additionally isolated *Acinetobacter schindleri *(*A. schindleri*); given the clinical context and absence of typical virulence characteristics, this was considered a probable co-colonizer or contaminant, and specific anti-*Acinetobacter* therapy was not separately initiated. Blood cultures did not yield *B. pseudomallei*, which is consistent with the recognized low sensitivity of blood cultures in musculoskeletal melioidosis [3]. No intra-abdominal abscesses were identified on imaging.

Treatment and course

On the day following the HRCT thorax, the patient underwent emergency right distal thigh abscess drainage, debridement, and bone biopsy under spinal anesthesia. The intraoperative and immediate postoperative period were uneventful. Following microbiological confirmation of *B. pseudomallei*, the patient was commenced on intravenous meropenem as the intensive phase antibiotic of choice for severe melioidosis.

During the hospital stay, the patient developed progressive pain and swelling in the left knee and heel. He also developed involuntary passage of stools attributed to reduced anal sphincter tone, raising concern for evolving spinal cord involvement in the context of the cervical MRI findings. An urgent neurosurgical consultation was obtained. In view of the suboptimal initial cervical MRI and the neurological symptom, contrast-enhanced MRI of the spine was recommended; however, clinical stabilization with antibiotic therapy was prioritized given the patient’s overall condition. Septic arthritis of the left knee confirmed on MRI prompted an emergency left knee arthrotomy, one week after the right distal thigh abscess drainage, under general anesthesia. Postoperatively, the patient was transferred to the intensive care unit (ICU) in view of persistent tachycardia.

Intravenous co-trimoxazole was subsequently added to the antibiotic regimen as an adjunct alongside continued meropenem, consistent with published experience in severe melioidosis [3]. Gradual clinical improvement was noted, with subsidence of left knee pain and left ankle/heel pain. Persistent fever spikes and tachycardia, however, continued during the index admission.

At discharge, the surgical wounds (right thigh and left knee) were clean and healing. The patient was actively moving both lower limbs, with distal pulses well palpable. He was discharged with instructions for continuation of the oral eradication phase antibiotic (trimethoprim-sulfamethoxazole) and close outpatient follow-up.

## Discussion

Melioidosis is caused by *B. pseudomallei*, a Gram-negative, aerobic, non-motile bacillus. First described by Whitmore and Krishnaswami in 1912 in Burma, the organism is a free-living environmental saprophyte found in soil and water in endemic tropical regions [7]. India has increasingly been recognized as an endemic region, with cases predominantly reported from the coastal and southern states, including Tamil Nadu, Kerala, and Karnataka [8].

The disease is often called “the great mimicker” because its clinical manifestations are indistinguishable from a host of other bacterial, tuberculous, and fungal infections [4]. In the present case, the initial presentation of fever, osteomyelitis, and abscess formation raised differential diagnoses including pyogenic osteomyelitis, tuberculosis, and hematogenous septic arthritis. The diagnostic gold standard remains bacterial culture of pus, blood, sputum, or tissue, as was the case here with pus yielding *B. pseudomallei* [3].

Diabetes mellitus is the single most important risk factor for melioidosis, present in up to 37-60% of cases in endemic series [3,9]. Our patient had newly diagnosed type II diabetes mellitus, which likely predisposed him to hematogenous dissemination and multifocal skeletal seeding of the organism. The mechanism of skeletal melioidosis is thought to involve hematogenous spread from a primary focus, with direct percutaneous inoculation being a less common route [6].

Musculoskeletal melioidosis is an established but infrequent presentation, accounting for 4-14% of cases in large series. The knee joint is the most commonly affected joint, followed by the ankle, hip, and shoulder [6]. Multifocal bone involvement, as seen in our patient - encompassing the right distal femur, bilateral knees, left calcaneum, talus, distal tibia, and cervical vertebrae - represents a particularly severe and diagnostically challenging presentation. Vertebral melioidosis has been described in isolated case reports and is considered rare. The possibility of spinal osteomyelitis (C2-C6) in this patient, while requiring contrast-enhanced MRI confirmation, is consistent with hematogenous dissemination from the primary skeletal foci. The development of reduced anal sphincter tone in this clinical context further raises the concern of spinal cord involvement and warrants urgent neuroimaging and specialist input.

Pulmonary involvement in the form of multiple cavitating nodules consistent with septic emboli, identified on HRCT, further reflects the disseminated nature of the infection. This finding, in conjunction with multifocal skeletal disease, places this case in the category of disseminated melioidosis, a form associated with significant mortality if not recognized and treated early [3].

The management of melioidosis follows a two-phase antibiotic strategy. The intensive phase employs intravenous meropenem or ceftazidime for a minimum of 10-14 days (and up to four to eight weeks in severe or disseminated disease), followed by an eradication phase with oral trimethoprim-sulfamethoxazole for three to six months [3,10]. In our patient, meropenem was initiated following culture confirmation, and intravenous co-trimoxazole was added as an adjunct during the intensive phase, as has been described in severe disseminated disease [3,11]. Surgical debridement of all accessible septic foci is an integral component of treatment, as demonstrated by the right thigh debridement and left knee arthrotomy in this case [11].

A notable finding in this case was the co-isolation of *A. schindleri* from bone marrow culture. This organism is an infrequent clinical isolate of generally low virulence, and its recovery in this setting was attributed to probable co-colonization or contamination rather than true co-infection. Clinical response to meropenem - which also provides coverage for Acinetobacter species - was consistent with this interpretation.

From a laboratory perspective, clinicians and microbiologists in endemic regions must maintain a heightened awareness of *B. pseudomallei*. The organism may be misidentified by automated identification systems, and handling requires biosafety level 3 (BSL-3) containment procedures [3]. Laboratories should be explicitly alerted to the clinical suspicion of melioidosis when specimens are submitted, particularly for deep-seated pus and bone biopsy material, to ensure appropriate culture conditions and safety measures.

A significant challenge in this case was the diagnostic delay attributable to the protean nature of the infection and its initial resemblance to common pyogenic osteomyelitis. This underscores the importance of sending deep-seated pus and bone biopsy specimens for extended aerobic culture in regions where melioidosis is endemic, or in patients with diabetes presenting with atypical or treatment-refractory musculoskeletal infections [8].

## Conclusions

This case illustrates the diagnostic and therapeutic complexity of disseminated skeletal melioidosis in a newly diagnosed diabetic patient. The simultaneous involvement of multiple skeletal sites - right distal femur, bilateral knee joints, left ankle complex (distal tibia, calcaneum, and talus), and cervical vertebrae - combined with pulmonary septic emboli and possible spinal cord involvement, represents one of the more severe multifocal presentations of this disease. Melioidosis must be considered in the differential diagnosis of any patient presenting with multifocal osteomyelitis or septic arthritis in an endemic region, particularly in the presence of diabetes mellitus. Early deep tissue culture, prompt initiation of targeted antibiotic therapy with intravenous meropenem, and timely surgical debridement of accessible foci are the cornerstones of management. Heightened awareness among clinicians and microbiologists is essential to reduce diagnostic delay and improve outcomes in this potentially life-threatening infection.
